# Therapeutic Drug Monitoring of Mycophenolic Acid as a Precision Medicine Tool for Heart Transplant Patients: Results of an Observational Pharmacokinetic Pilot Study

**DOI:** 10.3390/pharmaceutics14061304

**Published:** 2022-06-20

**Authors:** Francesco Lo Re, Jacopo Angelini, Sandro Sponga, Chiara Nalli, Antonella Zucchetto, Jessica Biasizzo, Ugolino Livi, Massimo Baraldo

**Affiliations:** 1 Clinical Pharmacology and Toxicology Institute, University Hospital Friuli Centrale ASU FC, 33100 Udine, Italy; francesco.lore@asufc.sanita.fvg.it (F.L.R.); jacopo.angelini@asufc.sanita.fvg.it (J.A.); 2 Department of Medical Area (DAME), University of Udine (UNIUD), 33100 Udine, Italy; sandro.sponga@uniud.it (S.S.); ugolino.livi@uniud.it (U.L.); 3 Department of Cardiothoracic Surgery, University Hospital Friuli Centrale ASU FC, 33100 Udine, Italy; chiara.nalli@asufc.sanita.fvg.it; 4 Scientific Directorate, Centro di Riferimento Oncologico di Aviano (CRO), Istituto di Ricovero e Cura a Carattere Scientifico (IRCCS), 33081 Aviano, Italy; azucche@libero.it; 5 Institute of Clinical Pathology, University Hospital of Friuli Centrale ASU FC, 33100 Udine, Italy; jessica.biasizzo@asufc.sanita.fvg.it

**Keywords:** therapeutic drug monitoring, pharmacokinetic interactions, heart transplant, precision medicine, clinical practice

## Abstract

In the clinical practice management of heart transplant (HTx), the impact of calcineurin inhibitors co-administration on pharmacokinetics (PKs) of mycophenolic acid (MPA), mycophenolate mofetil (MMF) active drug, is not adequately considered. This retrospective study investigated full MPA-PK profiles by therapeutic drug monitoring (TDM) in 21 HTx recipients treated with MMF combined with cyclosporine (CsA) or tacrolimus (TAC) at a median time of 2.6 months post-transplant. The two treatment groups were compared. We described the main MPA-PK parameters in patients developing acute cellular rejection (ACR) and those who did not. Median dose-adjusted MPA-trough levels and MPA-AUC_0–12h_ were higher in patients co-treated with TAC than with CsA (*p* = 0.0001 and *p* = 0.006, respectively). MPA-C_max_ and T_max_ were similar between the two groups, whereas the enterohepatic recirculation biomarker of MPA (MPA-AUC_4–12h_) was higher in the MMF and TAC group (*p* = 0.004). Consistently, MPA clearance was higher in the MMF and CsA group (*p* = 0.006). In total, 87.5% of ACR patients were treated with MMF and CsA, presenting a lower MPA-AUC_0–12h_ (*p* = 0.02). This real-world study suggested the CsA interference on MPA-PK in HTx, evidencing the pivotal role of MPA TDM as a precision medicine tool in the early phase after HTx. A prospective study is mandatory to investigate this approach to HTx clinical outcomes.

## 1. Introduction

Mycophenolate mofetil (MMF) represents a cornerstone for the treatment of heart transplant (HTx) [1]. Standard immunosuppressive maintenance protocols include its administration combined with calcineurin inhibitors (CNIs), such as cyclosporine (CsA) or tacrolimus (TAC), and corticosteroids.

After oral administration, MMF is rapidly metabolized to mycophenolic acid (MPA), which is a selective and noncompetitive inhibitor of inosine-5′-monophosphate dehydrogenase (IMPDH) that causes the arrest of the proliferation of T- and B-cells [2]. When orally administered, the time to reach MPA plasma maximum concentration (T_max_) occurs after approximately 1–2 h. Frequently, MPA exhibits a secondary peak due to enterohepatic recirculation (EHC), occurring 6–12 h after drug administration [2]. Indeed, uridine diphosphate glucuronosyltransferase (UDP-GT) enzyme family metabolized MPA into its main inactive metabolite 7-O-MPA-glucuronide (MPAG), which is mainly excreted in the urine and into the bile by multi-drug resistance protein 2 (MRP-2). Hence, MPAG can be hydrolyzed into MPA by gastrointestinal flora and then reabsorbed by EHC [2]. Since CsA inhibits MRP-2, in the case of MMF and CsA co-administration MPA secondary peak is suppressed [2]. In renal transplant patients, Cattaneo et al. evidenced a decrease in MPA concentrations, occurring 4–12 h from MMF administration, as suggested by the corresponding biomarker represented by the area under the concentration–time curve from 4 to 12 h (AUC_4–12h_) [2,3,4]. Consistently CsA administration increases MPA clearance (Cl) [5].

From a pharmacokinetic (PK) point of view, MPA exposure, in terms of its area under the 12 h concentration–time curve (AUC_0–12h_), represents the major prognostic PK parameter for this immunosuppressive treatment [6]. MPA-AUC_0–12h_ is characterized by a relevant inter- and intra-subject variability [7]. It has been reported a >10-fold variation in dose-adjusted MPA-AUC_0–12h_ among patients in heart and renal transplants [8]. Some authors also suggested a relationship between MPA plasma concentration and incidence of cardiac rejection [9,10]. For this reason, the therapeutic drug monitoring (TDM) of MPA is recommended to maximize the efficacy of treatment, especially in the first period after transplant and in patients with high immunological risk [6].

A therapeutic MPA-AUC_0–12h_ target, ranging from 30 to 60 mg·h/L, was prospectively validated in renal transplant patients [6,11]. In the Htx setting, similar thresholds were identified [12,13], although supported by low strength of recommendation and scarce quality of evidence [6].

This retrospective observational monocentric pilot study was designed to compare the effect of CsA or TAC administration on MPA-PK in a cohort of HTx recipients. Patients were treated according to the standard clinical practice of our University Hospital Center. In particular, we primarily sought to compare the 12-h MPA-PK profiles in both groups in the first period after transplant and to examine the effect of CsA on MPA EHC.

Secondarily, we only exploratively described the main MPA-PK parameters in patients reporting acute cellular rejection (ACR) and in those who did not (NACR).

## 2. Materials and Methods

### 2.1. Patients

In total, 21 adult patients who underwent primary HTx were included in our analysis. All patients had been previously treated at the University Hospital of Udine following the internal protocol with MMF and CsA (Group 1) or MMF and TAC (Group 2) and prednisone [14]. From 1 January 2011 to 31 December 2019, TDM of immunosuppressive drugs was performed for each patient during a minimum follow-up period of 12 months. All consecutive HTx recipients in the study period who met these criteria were included in the analysis. Patients treated with prokinetic drugs, resins, or other drugs interfering with MPA-PK, except for prednisone, were excluded. Patients treated with other immunosuppressive agents than MMF, CsA, and TAC or subjects with relevant missing data in the clinical records or without informed consent for clinical, epidemiological research, training, and study of pathologies were excluded from the study.

CsA and TAC were administered according to the clinical condition and renal function. CsA and TAC doses were adjusted to reach different blood concentration targets, according to our clinical practice adapted from literature: 150–250 ng/mL and 100–200 ng/mL for CsA; 10–15 ng/mL and 5–10 ng/mL for TAC, <3 months and >3 months post-transplant, respectively [15,16].

MMF was administered with a median dose of 750 mg twice daily, and dose-adjustments were performed according to blood leucocyte count, clinical conditions, drug tolerability, or adverse effects. The TDM of MPA was executed at a median time of 2.6 months post-transplant, setting an AUC_0–12h_ target range at 30–60 mg·h/L [6].

### 2.2. Study Design and Pharmacokinetic Measurements

This is a pilot, observational, retrospective cohort study.

Pharmacological, hematological, and biochemical analyses were performed according to routine clinical practice. PK analyses were required when the expected steady state was reached for all drugs. In particular, PK analysis consisted of the 12 h MPA-PK profile, CsA, and TAC pre-dose measurement. Patients were asked to take their usual morning dose of MMF, CsA, or TAC after having a standard meal.

For the 12 h MPA-PK profile, written informed consent had been obtained, and 8 venous blood samples had been taken at 0 (pre-dose), 0.5, 1.25, 2, 4, 6, 8, and 12 h after MMF morning dose. Each patient executed a single PK profile one time only. Separation of plasma was performed immediately in a centrifuge at 4 °C. Plasma MPA concentrations were measured by high-pressure liquid chromatography with UV detector (HPLC/UV) using a reference method published in literature [17]. Our laboratory reported the following parameter for the HPLC/UV method used: limit of detection: 0.1 µg/mL; linearity between 0.1 and 40 µg/mL (coefficient of determination, R^2^: 0.9988); intra-batch imprecision expressed as coefficient of variation (CV): 3.15%, 1.55%, and 1.76% at MPA plasma concentrations of 1.5, 5.0, 15.0 µg/mL, respectively; inter-batch imprecision (CV): 3.41%, 3.21%, and 1.92% at MPA plasma concentrations of 1.5, 5.0, 15.0 µg/mL, respectively; overall inaccuracy (% bias) of the procedure: ranged from 8.7% to 13.6%. TAC and CsA trough levels were determined in whole blood for all patients by an affinity column-mediated immunoassay (ACMIA) method using a Dimension Vista 1500^®^ Analyzer (Siemens Healthcare Diagnostics, Berlin, Germany). Our laboratory reported the following parameter CsA: assay range (25–500 ng/mL); linearity from 25 to 200 ng/mL; limit of detection 25 ng/mL; within-run precision (CV): 5.2%, 4.3%, and 4.3% at CsA concentrations of 65, 151, 400 ng/mL, respectively; within laboratory precision (CV): 8.2%, 5.4%, and 4.8% at CsA concentrations of 65, 151, 400 ng/mL, respectively. For TAC the parameter reported were the followings: assay range (1–30 ng/mL); linearity from 1 to 30 ng/mL; limit of detection 0.7 ng/mL; within-run precision (CV): 3.8%, 2.3%, and 3.1% at TAC concentrations of 4.4, 11.4, 27.4 ng/mL, respectively; within laboratory (CV): 6.9%, 4.5%, and 5.1% at TAC concentrations of 4.4, 11.4, 27.4 ng/mL, respectively.

MPA PK was evaluated by a non-compartmental model after oral administration using the PK solver software^®^ [18], according to similar studies in the literature [4,19,20]. MPA-AUC_0–12h_ was calculated by means of the linear trapezoidal rule.

According to Cattaneo et al. [4], we investigated the possible impact of TAC or CsA on MPA bioavailability, taking into consideration the area under the concentration–time vurve from 0 to 2 h (AUC_0–2h_), as a surrogate marker of absorption, the MPA peak plasma concentration (C_max_) and the T_max_. Furthermore, we evaluated the MPA-AUC_4–12h_ as a surrogate marker of EHC. MPA apparent clearance (Cl/F) was evaluated by this formula for both groups:

(1) $$\mathrm{MPA}\;\mathrm{Cl}/F=\left(\mathrm{MPA}\;\mathrm{morning}\;\mathrm{dose}\right)⁄\left(\mathrm{MPA}\;{\mathrm{AUC}}_{0–12h}\right)\;$$

MPA-PK parameters were assessed and compared between patient groups.

Secondarily, as explorative analysis, we described MPA-AUC_0–12h_ values, all the drug serum concentration sampling points, C_max_ absolute and dose-adjusted values in ACR and NACR patients.

To exclude the confounding factor of different MMF drug doses, MPA-PK parameters were normalized for the daily MMF dose according to the following formula [4]:

(2) MPA PK parameter dose−adjusted =(MPA PK parameter)/(MMF daily dose)

### 2.3. Rejection Assessment

The incidence of ACR was evaluated by endo-myocardial biopsies executed as per standard clinical practice [14] and classified according to the International Society for Heart and Lung Transplantation (ISHLT) standardized grading method [21]. ACR was defined as an ISHLT grade greater than or equal to 1 at the time of observation after transplant on endomyocardial biopsy specimens.

### 2.4. Statistical Analysis

Categorical variables were presented as absolute value and relative frequency (percentage) and continuous variables as the median and interquartile range (IQR). Normality was assessed using Shapiro–Wilk test. Categorical variables were compared using the Fisher’s exact test, whereas continuous variables were compared using Mann–Whitney test, according to the distribution of the data. The non-parametric Mann–Whitney test was used to compare MPA PK values between the study groups and between male and female patients. *p*-values of a 2-sided test lower than 0.05 were considered statistically significant. Analyses were performed using MedCalc (Statistical Software version 20.106, Ostend, Belgium; http://www.medcalc.org accessed on 1 March 2022). Correlation among multiple variables with nonparametric distribution data was performed by Spearman test, considering r > 0.6 as strong correlation.

The ratio between the median value of each MPA-PK parameter we measured in Group 1 and Group 2 was used to estimate the degree of the differences between the two groups according to the following formula:

(3) RATIO= median PK parameter Group 2/Group 1

The same evaluation was assessed among NACR and ACR patients as follows:

(4) RATIO= median PK parameter NACR patients/ACR patients

## 3. Results

### 3.1. Patients Characterization

Twenty-one patients were included in the analysis, fourteen men (67.0%) and seven (33.0%) women. Twelve of them (57.1%) were included in Group 1, whereas nine (42.9%) were in Group 2. Treatment groups were balanced in terms of relevant clinical characteristics, as shown in Table 1.

### 3.2. Analysis of Patients’ Primary Outcome: PK Analysis

The results of the 12-h MPA-PK analysis performed at a median time of 2.8 months (IQR; 1.1–6.7 months) in Group 1 and at 2.6 months post-transplant (IQR: 2.3–5.6 months) in Group 2 are shown in Table 2.

Absolute and dose-adjusted MPA-C_0_ values were significantly higher in Group 2 (*p* = 0.001), and the ratio between median values of the two groups was 2.35 and 2.70, respectively.

The T_max_, absolute, and dose-adjusted MPA-C_max_ and AUC_0–2h_ results did not show a statistically significant difference between the two groups of treatment.

**Figure 1:**
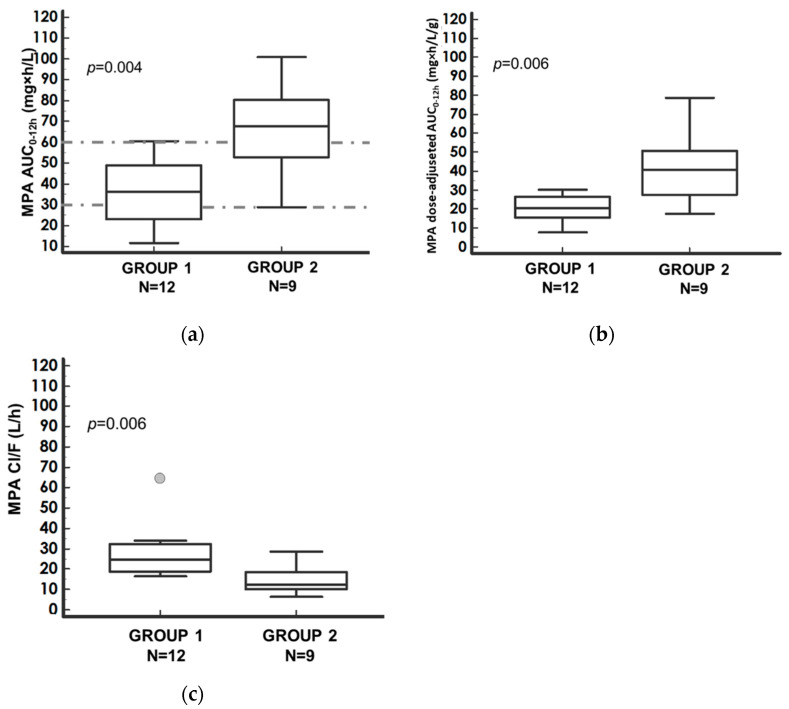
Comparison of (**a**) MPA–AUC_0–12h_; (**b**) dose-adjusted MPA–AUC_0–12h_; (**c**) MPA–CL/F, between Group 1 (MMF + CsA group) and Group 2 (MMF + TAC group) and corresponding *p*-value of the Mann–Whitney test. Median values with the corresponding 95% confidence intervals (bars). Dots represent the outliers. In (**a**), the interval between the dotted lines represents the therapeutic range (30–60 mg·h/L). Nomenclature: AUC_0–12h_—area under the 12 h concentration–time curve; Cl/F—apparent clearance; CsA—cyclosporine; MPA—mycophenolic acid; MMF—mycophenolate mofetil; TAC—tacrolimus.

MPA-AUC_0–12h_ absolute and dose-adjusted values were significantly higher in Group 2 (*p* = 0.04; *p* = 0.06, respectively) as shown in Figure 1a,b, and the ratio between median values were 1.88 and 2, respectively (Table 2). On the other hand, MPA Cl/F (L/h) in the Group 1 was higher than in Group 2 (*p* = 0.006) (Figure 1c).

**Figure 2:**
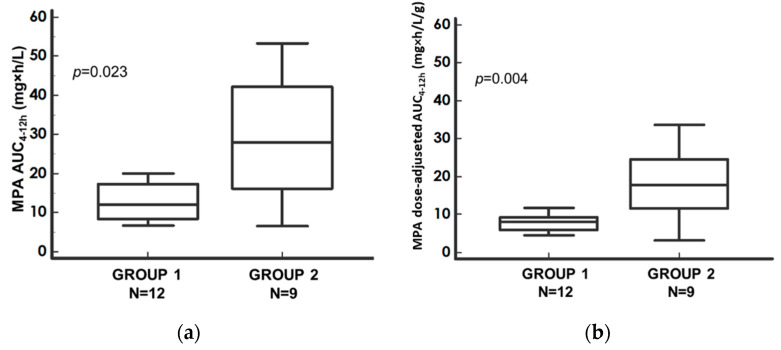
Comparison of (**a**) MPA–AUC_4–12h_; (**b**) dose-adjusted MPA–AUC_4–12h;_ between Group 1 (MMF + CsA group) and Group 2 (MMF + TAC group) and corresponding *p*-value of the Mann–Whitney test. Median values with the corresponding 95% confidence intervals (bars). Dots represent the outliers. Nomenclature: AUC_4–12h_—area under the 4–12-h concentration–time curve; CsA—cyclosporine MPA—mycophenolic acid; MMF—mycophenolate mofetil; TAC—tacrolimus.

The main difference in the MPA-PK profiles between the two immunosuppressive treatments was observed at the MPA-AUC_4–12h_. In Group 1, MPA absolute and dose-adjusted AUC_4–12h_ were significantly reduced compared to Group 2 (*p* = 0.023, *p* = 0.0036, respectively), as shown in Figure 2a,b. The absolute and dose-adjusted MPA-AUC_4–12h_ ratios between Group 2 and Group 1 median values were 2.30 and 2.19, respectively (Table 2).

We investigated whether other parameters such as age, sex, renal function, drug clearance, weight and BMI could interfere with the PK differences observed, according to cotreatments, but no correlations were found by Spearman Correlation (r < 0.6) and Mann-Whitney tests (*p* > 0.05). It was highlighted only a difference in terms of the MPA–AUC_0–2h_ among female and male patients (median values: 25.9; IQR: 15.8–29.4 vs. 13.7; IQR: 4.6–17.1 respectively; *p* = 0.03).

### 3.3. Descriptive Analysis of Patients Reporting Acute Cellular Rejection

Out of twenty-one treated patients, eight (38.1%) experienced ACR (six males and two females). Their daily median MMF dose was 1750 mg (IQR: 1375.0–2000.0), corresponding to 24.1 mg/kg/day (IQR: 19.4–30.7). Seven of them (87.5%) were co-treated with CsA, and we investigated the impact of CNIs co-treatments on ACR (Table 3).

The grade of rejection ranged from mild to severe (grade 1R–3R, as shown in Table 4). It occurred at a median time of 3.0 months (IQR: 2.5–10.1) after HTx.

The TDM was performed at a median time of 1.04 months (IQR: −1.91–3.36) from ACR assessment. To investigate the presence of potential bias due to a different intake of CNIs, we compared NACR and ACR patients according to the CsA or TAC daily dose co-treatment. No differences in terms of daily dose for each CNIs were found with regard to CsA (*p* = 0.56), with a median CsA daily dose equal to 200 mg/kg/day (IQR: 168.75–212.5) among NACR vs. 200 mg/kg/day (IQR: 162.5–343.75) among ACR patients. Due to just a single case of ACR in the TAC-treated group, the test could not be performed.

MPA PK parameters in NACR and ACR patients are reported in Table 5.

**Figure 3:**
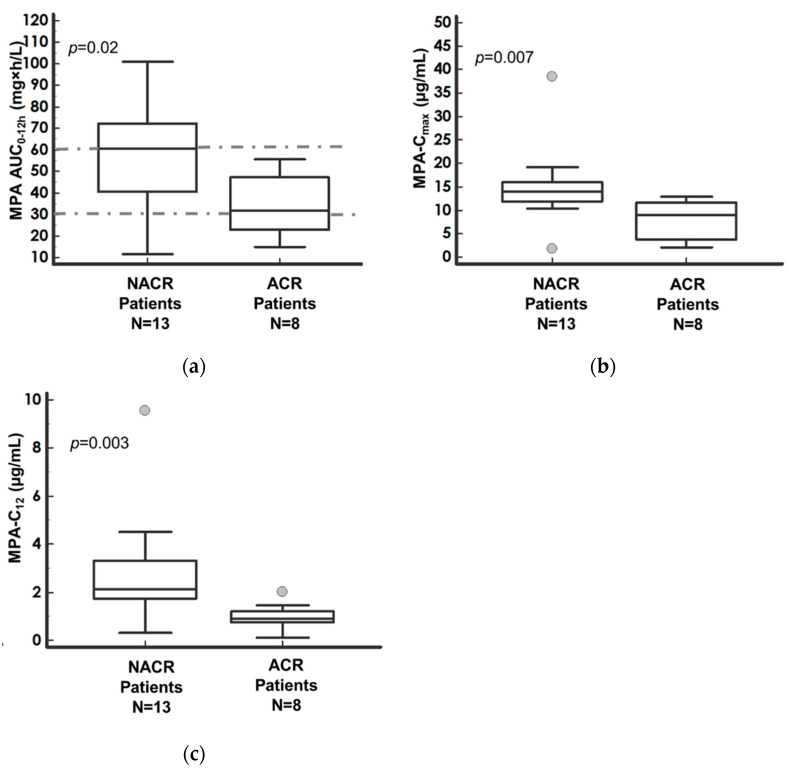
Comparison of (**a**) MPA–AUC_0–12h_, (**b**) MPA–C_max_, and (**c**) MPA–C_12_ between NACR and ACR patients and corresponding *p*-value of the Mann–Whitney test. Median values with the corresponding 95% confidence intervals (bars). In (**a**), the interval between the dotted lines represents the therapeutic range (30–60 mg·h/L). Dots represent the outliers. Nomenclature: AUC_0–12h_—area under the 12-h concentration–time curve; C_max_—peak drug plasma concentration; C_12_—12 h post-dose drug plasma concentration, ACR pts—patients who developed acute cell rejection; NACR pts—patients who did not develop acute cell rejection.

MPA-AUC_0–12h_ median value in NACR patients was higher than in ACR patients (*p* = 0.02), as shown in Figure 3a, and the ratio between median values was 1.9 (Table 5). These results were also confirmed for the dose-adjusted parameter (Table 5). No statistically significant difference in MPA-C_0_ between the two groups of patients was found (Table 5). On the contrary, NACR recipients presented a higher MPA-C_max_ (*p* = 0.007), as shown in Figure 3b, than ACR, and the ratio between the median values was 1.6 (Table 5). Significantly increased values were evidenced for MPA-C_12h_ (*p* = 0.003), as shown in Figure 3c, and the ratio between NACR and ACR recipients was 2.4 (Table 5).

All these differences were confirmed by the analysis of the corresponding dose-adjusted parameters, as shown in Table 5.

## 4. Discussion

MPA-PK profile can be influenced by the CNIs co-treatment, as evidenced by the effect of CsA on MPA EHC [6]. Due to the critical conditions of patients in the early post-HTx period [15] and the possible pharmacological interaction of the co-administration of immunosuppressive agents, the TDM of MPA could play a pivotal role in the context of precision medicine in clinical practice. In this study on HTx recipients treated with MMF, we found marked differences in MPA-AUC_0–12h_, which were influenced by the co-administered CNI, resulting in a two-fold higher exposure in patients co-treated with TAC. No influences of CsA or TAC were observed on both MMF adsorption and bioavailability, as supported by our data on MPA AUC_0–2h_, C_max_ and T_max_. On the contrary, we evidenced a higher dose-adjusted MPA-AUC_4–12h_ in Group 2 than in Group 1 and a higher MPA-Cl/F in Group 1. These results suggest the inhibition of EHC by CsA in HTx recipients. The higher MPA trough levels observed in Group 2 (Table 2) could be explained by the interference of CsA on MPA EHC since it frequently occurs from 6 to 12 h after MMF administration. Considering the confounding factor of MMF administered dose, MPA-PK parameters were adjusted for daily dose. This approach presumed linearity between MMF doses and MPA exposure. By comparing the absolute and dose-adjusted MPA PK parameters, linearity between dose and concentration was confirmed, as suggested by Cattaneo et al. in renal transplant patients [4].

These data are aligned to a similar study involving 62 HTx recipients, where patients co-treated with CsA and MMF showed higher MPA exposure and pre-dose levels than patients co-treated with sirolimus, confirming the effect of CsA on MPA exposure as evidenced in our study [22]. Previous studies have already shown that CsA could influence MPA-AUC_0–12h_ affecting MPA EHC [2] and MPA-Cl [5]. Nevertheless, this PK interaction has been well documented in renal transplant recipients [4,22,23], whereas there is a paucity of data from studies investigating not-renal transplant recipients, where renal clearance and drugs half-life should be less involved.

The impact of MPA TDM on HTx has been less studied, differently from renal transplant. Two reviews resumed the previous clinical trials in the HTx setting, investigating a relationship between MPA-PK parameters and therapy outcomes [24,25]. Although the prevention of ACR in HTx patients is not totally defined, it has been suggested that MPA-AUC_0–12h_ monitoring represents a more effective strategy to prevent rejection than a single-time point model [24]. For this reason, in this pilot study, we exploratively described the PK parameters in NACR and ACR patients, obtaining results in terms of absolute and dose-adjusted MPA-AUC_0–12h_ (*p* < 0.03), which are aligned with the main relevant data in the literature. De Nofrio et al. showed in 38 Htx recipients that patients with a grade 2 or 3 rejection had a lower mean total MPA-AUC_0–12h_ (26.1 ± 6.6 vs. 42.8 ± 14.0 mg·h/L, *p* < 0.08), while no effect of MPA trough level was found [26]. According to the last Consensus Report by the International Association of Therapeutic Drug Monitoring and Clinical Toxicology on MMF, different thresholds have been proposed [6]. Figurski et al. showed in 21 HTx patients that an MPA-AUC_0–12h_ < 36.2 mg·h/L in the first two weeks post-transplant was associated with a higher risk of ACR, with high sensitivity (0.8) and specificity (0.8), although the statistical significance was not reached [13]. Woillard et al. identified for MPA-AUC_0–12h_ a threshold of 50 mg·h/L, below which there was a higher risk of rejection, stratifying patients for CNI exposure (*p* = 0.002) [12].

In this study, since no MMF dose changes occurred, we supposed that ACR patients were less exposed to immunosuppressive maintenance treatment than NACR patients. Due to the higher prevalence of ACR recipients in Group 1 compared to Group 2, this clinical outcome could also be attributable to the influence of CsA on MPA-AUC_0–12h_. The Fisher exact test we performed to explore this hypothesis showed only a trend (*p*-value 0.067), probably due to a limited number of patients included in our analysis. Furthermore, we evidenced a statistically significant difference between NACR and ACR patients not only on MPA-C_max_ (*p* = 0.01) but also on MPA-C_12_ (*p* = 0.003), which previous studies demonstrated an acceptable correlation to the entire MPA-AUC_0–12h_ [7,27,28]. These data confirmed the strong relationship between total drug exposure and immunosuppressive efficacy. On the contrary, MPA-C_0_ does not statistically discriminate between ACR and NACR patients in our analysis, resulting in a weak surrogate marker of total drug exposure and efficacy. Hence, the estimation of MPA-AUC_0–12h_ by a limited sampling scheduled should be preferred to a pre-dose determination. Alternatively, if only one sample can be obtained, Kaczmareck et al. showed that MPA-C_12_ could be a better efficacy biomarker than MPA-C_0_ due to the scheduled sampling time strongly linked to dose administration [29].

In our analysis, we excluded possible PK interpretation biases such as the interference of glucocorticoids in MPA exposure and bilirubinemia. Glucocorticoids induce the expression of UDP-GT, the inactivating enzyme of MPA [2]. Nevertheless, all patients received the same mg/kg/day dose of prednisone, not representing a confounding variable. On the other hand, MPA-AUC_0–12_ could be affected by bilirubinemia, which was different in Group 1 and Group 2 [23]. High plasmatic bilirubin levels could displace MPA from albumin binding sites, thus affecting its total exposure [30]. We excluded this possible influence since our patients did not show clinically relevant high levels of plasmatic bilirubin, and hyperbilirubinemia does not significative affect the free active MPA concentration [27]. We did not consider the different baseline platelet levels in Group 1 and Group 2 since no data in the literature show possible influence on immunosuppressive treatments.

Due to the small number of patients and the retrospective and observational nature of this study, MPA-AUC_0–12h_ was measured by a single PK profile analysis per patient which was not executed at the same time for all the enrolled patients and not strictly close to ACR, as it was executed by clinical practice, not following a specific study protocol. Hence, no conclusive results can be obtained. However, we assumed that MPA-PK parameters were most likely stable during the observational time since physicians did not observe any clinical conditions requiring a dose adaptation of the standard immunosuppressive regimen. Furthermore, MPA PK prospective studies on heart and renal transplants did not show relevant variability of these parameters during the first year of treatment, considering both absolute and dose-adjusted data [4,13]. This misalignment between PK measurements and clinical assessments could represent the major source of bias that prevented us from assessing whether there was a meaningful clinical relation between ACR and low MPA exposure. Nevertheless, the PK parameters we found in our analyses were consistent with other similar PK studies in terms of (a) increased Cl/F in Htx recipients co-treated with CsA compared to TAC, (b) two-fold increased AUC_0–12h_ in TAC than in CsA co-treated patients, and (c) identification of a relevant MPA-EHC in TAC co-treated recipients [4,20]. Another source of bias in this study could be related to the overall study group composition, mainly represented by men, whereas Group 2 included a high percentage of women. However, MPA-PK generally is not influenced by gender [2,31], although it was recently suggested for MPA and MPAG PK in stable renal transplant recipients receiving enteric coated mycophenolate sodium combined with TAC [32]. Interestingly, in our analysis, we did not observe any relevant PK differences, except for the MPA-AUC_0–2h_, suggesting a better absorption of MMF in female patients than in male patients.

Due to the aforementioned limitations, our findings cannot be transferred into clinical practice. A prospective study with homogeneous scheduled serial measurements per subject, from the enrolment up to the development of rejection, is required.

## 5. Conclusions

This pilot study evidenced that TAC or CsA co-administration could affect MPA exposure in HTx recipients. This effect is attributable to MPA EHC suppression and consequent MPA-Cl augmentation by CsA. These results have to be prospectively validated in order to support a precision medicine approach in routine clinical practice. MPA TDM represents a relevant supporting tool for clinicians, especially in the early post-transplant period, when MMF is administered at a fixed dose. In this context, the estimation of the MPA-AUC_0–12_ by a few sampling points (i.e., limited sampling strategies) is a more effective approach than a single sample point model (i.e., C_0_). This method could optimize MMF efficacy and minimize adverse effects, especially when recipients are switched from CsA to TAC or vice versa. The findings of our explorative study can be used in order to design a future prospective trial aiming to identify the optimal thresholds for the main clinically relevant MPA-PK parameters, stratifying patients for the co-administered CNI after HTx.

All these data further support the MPA TDM employment in clinical practice.

## Figures and Tables

**Table 1:** Patients’ baseline demographical and clinical data are reported as overall and according to the immunosuppressive treatment.

Parameter	Total	Group 1	Group 2	
	Median (IQR)	Median (IQR)	Median (IQR)	*p*-Value
Number of patients (N, %)	21 (100%)	12 (57.1%)	9 (42.9%)	-
Males (N, %)	14 (67%)	10 (83%)	4 (44%)	0.09 ^a^
Age (years)	56.0 (42.0–62.9)	58.3 (49.8–60.5)	43.1 (41.0–65.6)	0.57
MMF dose (mg/day)	1500 (1500–2000)	1500 (1500–2000)	1500 (1500–2000)	1.00
MMF dose (mg/kg/day)	26.3 (20.5–29.7)	24.7 (19.9–28.1)	26.3 (20.8–29.7)	0.72
Post-transplant time (months)	2.6 (1.9–6.1)	2.8 (1.1–6.7)	2.6 (2.3–5.6)	0.67
BMI (Kg/m^2^)	22.7 (19.9–28.7)	22.7 (19.9–29.2)	22.7 (20.2–26.8)	0.86
RBCs (×10^6^/μL)	3.9 (3.6–4.2)	4.0 (3.6–4.4)	3.8 (3.5–4.0)	0.23
Hb (g/dL)	11.6 (10.3–12.8)	11.6 (10.5–12.5)	11.6 (10.3–12.8)	0.7
WBCs (×10^3^/μL)	7.9 (6.2–9.2)	8.0 (6.7–10.1)	6.5 (5.2–8.5)	0.21
Neutro (×10^3^/μL)	5.3 (3.9–7.4)	6.1 (4.4–7.7)	4.3 (3.7–6.2)	0.14
Lymph (×10^3^/μL)	1.0 (0.6–1.5)	0.9 (0.5–1.3)	1.1 (0.9–1.5)	0.41
Mono (×10^3^/μL)	0.6 (0.5–0.8)	0.6 (0.5–0.8)	0.7 (0.6–0.7)	0.43
Eos (×10^3^/μL)	0.09 (0.02–0.12)	0.09 (0.02–0.11)	0.09 (0.03–0.12)	0.86
Bas (×10^3^/μL)	0.04 (0.01–0.06)	0.05 (0.02–0.05)	0.04 (0.01–0.07)	0.91
Plt (×10^3^/μL)	219.0 (195.0–299.0)	206.0 (173.0–214.5)	255.0 (221.0–311.0)	0.04 *
ALT (IU/L)	19.3 (17.0–30.0)	21.5 (17.8–31.0)	18.0 (17.0–19.3)	0.31
AST (IU/L)	19.0 (14.6–30.0)	19.5 (15.5–24.3)	19.0 (14.6–20.0)	0.52
Albumin (mg/dL)	42.4 (36.0–44.7)	37.5 (34.9–43.9)	44.4 (41.4–47.8)	0.05
Bilirubin (mg/dL)	0.7 (0.4–1.1)	1.0 (0.7–1.3)	0.4 (0.4–0.6)	0.01 *
CrCl (mL/min) ^b^	56.0 (48.0–80.0)	60.5 (37.0–83.8)	55.0 (52.0–68.0)	0.83
GFR (mL/min/1.73 m^2^) ^c^	60.0 (50.5–81.0)	61.5 (38.3–88.5)	60.0 (51.0–65.0)	0.89
Prednisone (mg/day)	15.0 (7.5–20.0)	12.5 (9.4–15.0)	20.0 (7.5–25.0)	0.26
Prednisone (mg/kg/day)	0.2 (0.1–0.3)	0.2 (0.1–0.2)	0.3 (0.1–0.4)	0.14
CsA dose (mg/day)	-	200.0 (168.8–250.0)	-	-
CsA dose (mg/kg/day)	-	2.9 (2.5–3.5)	-	-
CsA C_0_ (ng/mL)	-	184.6 (171.7–209.4)	-	-
TAC dose (mg/day)	-	-	4.0 (4.0–5.0)	-
TAC dose (mg/kg/day)	-	-	0.1 (0.1–0.1)	-
TAC C_0_ (ng/mL)	-	-	11.4 (9.9–12.0)	

Data are expressed as median and inter-quartile range (Q1–Q3), if not otherwise indicated. * Statistical difference between Group 1 and Group 2, *p* < 0.05 of Mann–Whitney test for continuous variables. ^a^ Fisher’s exact test for dichotomous variables. ^b^ Evaluated by Cockcroft–Gault adjusted for body weight. ^c^ Evaluated by CKD-EPI Equation; ALT—alanine aminotransferase; AST—aspartate aminotransferase; Bas—basophils; BMI—body mass index; C_0_—pre-dose drug concentration; CsA—cyclosporine; Eos—eosinophils; CrCl—creatinine clearance; GFR—glomerular filtration rate; Group 1—(MMF + CsA group); Group 2—(MMF + TAC group); Hb—hemoglobin level; IQR—interquartile range; Lymph—lymphocytes; Mono—monocytes; MMF—mycophenolate mofetil; Neutro—neutrophils; Plt—platelets; RBCs—red blood cells; TAC—tacrolimus; WBCs—white blood cells.

**Table 2:** Patients’ mycophenolic acid pharmacokinetics data in Group 1 and Group 2.

Parameter	Group 1		Group 2			
	Median	IQR	Median	IQR	*p*-Value	Ratio
MMF dose (mg/day)	1500	1500–2000	1500	1500–2000	1	1
MPA-C_0_ (μg/mL)	1.20	0.66–1.75	2.83	2.08–5.83	0.0014 *	2.35
MPA dose-adjusted C_0_ (μg/mL/g)	0.78	0.44–0.89	2.11	1.27–2.95	0.0014 *	2.70
MPA-C_max_ (μg/mL)	12.05	3.84–14.27	14.06	11.50–18.07	0.1769	1.16
MPA dose-adjusted C_max_ (μg/mL/g)	5.55	3.15–9.40	9.52	5.58–14.41	0.1021	1.72
T_max_ (min)	75	52.50–120.00	75	63.75–120.00	0.8806	1
MPA Cl/F (L/h)	24.63	18.85–32.12	12.28	10.18–18.34	0.0056 *	0.50
MPA-AUC_0–12h_ (mg·h/L)	36.05	22.95–47.85	67.60	52.75–80.30	0.0036 *	1.88
MPA dose-adjusted AUC_0–12h_ (mg·h/L/g)	20.40	15.57–26.58	40.73	27.50–50.63	0.0056 *	2.00
MPA-AUC_0–2h_ (mg·h/L)	13.80	3.67–19.55	16.31	14.97–29.23	0.1021	1.18
MPA dose-adjusted AUC_0–2h_ (mg·h/L/g)	7.28	3.63–10.51	12.92	7.94–21.67	0.1021	1.77
MPA-AUC_4–12h_ (mg·h/L)	12.12	8.48–17.40	27.91	16.09–42.17	0.0230 *	2.30
MPA dose-adjusted AUC_4–12h_ (mg·h/L/g)	8.10	5.84–9.25	17.77	11.67–24.46	0.0036 *	2.19

* Statistical difference between Group 1 and Group 2, *p* < 0.05 of Mann–Whitney test. AUC_0–2h_—area under the 0–2-h concentration–time curve; AUC_4–12h_—area under the 4–12-h concentration–time curve; AUC_0–12h_—area under the 12-h concentration–time curve; C_max_—peak drug plasma concentration; Cl/F—apparent clearance; Group 1—(MMF + CsA group); Group 2—(MMF + TAC group); IQR—interquartile range; MMF—mycophenolate mofetil; MPA—mycophenolic acid; Ratio—degree of the differences between the Group 2 vs. Group1; T_max_—time to reach the maximum drug plasma concentration.

**Table 3:** The impact of cyclosporine and tacrolimus co-treatments in patients with and without acute cellular rejection (ACR).

	CsA-Treated Patients N (%)	TAC-Treated Patients N (%)	Total N (%)	Fisher’s Exact Test *p*-Value
ACR Patients	7 (58.3)	1 (11.1)	8 (38.1)	0.067
NACR Patients	5 (41.7)	8 (88.9)	13 (61.9)	
Total	12 (100)	9 (100)	21 (100)	

ACR—acute cellular rejection; CsA—cyclosporine; NACR—not acute cellular rejection; TAC—tacrolimus.

**Table 4:** Patients’ acute cellular rejection (ACR) grading.

Parameter	N (%)
Patients reporting ACR ISHLT 1R (N, %)	5 (62.5%)
Patients reporting ACR ISHLT 2R (N, %)	2 (25.0%)
Patients reporting ACR ISHLT 3R (N, %)	1 (12.5%)

The percentage is referred to the total of ACR patients. ACR—acute cellular rejection; ISHLT—International Society for Heart and Lung Transplantation; 1R—mild grade; 2R—moderate grade; 3R—severe grade.

**Table 5:** Pharmacokinetics description in NACR and ACR patients.

Parameter	NACR Pts		ACR Pts			
	Median	IQR	Median	IQR	*p*-Value	Ratio
MMF dose (mg/day/kg)	26.32	20.14–29.46	24.09	18.21–34.04	0.86	0.91
MPA-AUC_0–12h_ (mg·h/L)	60.60	40.45–72.10	31.85	22.95–47.10	0.0248 *	1.90
MPA dose-adjusted AUC_0–12h_ (mg·h/L/g)	29.53	21.57–43.17	20.40	16.57–23.98	0.0298 *	1.45
MPA-C_0_ (µg/mL)	2.11	1.62–2.95	1.35	0.70–2.10	0.3106	1.60
MPA dose-adjusted C_0_ (µg/mL/g)	1.10	0.73–2.13	0.84	0.60–1.14	0.4257	1.31
MPA-C_max_ (µg/mL)	14.06	11.88–16.02	8.95	3.88–11.56	0.0074 *	1.60
MPA dose-adjusted C_max_ (µg/mL/g)	8.89	5.45–12.89	4.48	3.15–5.96	0.0357 *	1.98
MPA-C_12_ (µg/mL)	2.14	1.74–3.29	0.91	0.77–1.21	0.0030 *	2.36
MPA dose-adjusted C_12_ (µg/mL/g)	1.33	0.99–2.29	0.63	0.49–0.91	0.0059 *	1.60

* Statistical difference between NACR and ACR patients, *p* < 0.05 of Mann–Whitney test. Nomenclature: AUC_0–12h_—area under the 12-h concentration–time curve; ACR—acute cellular rejection; ACR pts—patients who developed ACR; NACR pts—patients who did not develop ACR; C_0_—pre-dose drug plasma concentration; C_12_—12 h post-dose drug plasma concentration; C_max_—peak drug plasma concentration; IQR—interquartile range; MPA—mycophenolic acid; Ratio—degree of the differences between the NACR vs. ACR patients.

## Data Availability

The data that support the findings of this study are available from the corresponding author upon reasonable request.
